# A single shell protein plays a major role in choline transport across the shell of the choline utilization microcompartment of *Escherichia coli* 536

**DOI:** 10.1099/mic.0.001413

**Published:** 2023-11-16

**Authors:** Jessica M. Ochoa, Philip Dershwitz, Mary Schappert, Sharmistha Sinha, Taylor I. Herring, Todd O. Yeates, Thomas A. Bobik

**Affiliations:** ^1^​ UCLA-Molecular Biology Institute, University of California, Los Angeles, USA; ^2^​ Roy J. Carver Department of Biochemistry, Biophysics and Molecular Biology, Iowa State University, Ames, IA, 50011, USA; ^3^​ UCLA-DOE Institute for Genomics and Proteomics, Los Angeles, USA; ^4^​ Department of Chemistry and Biochemistry, University of California, Los Angeles, USA

**Keywords:** choline, *E. coli 536*, microcompartment

## Abstract

Bacterial microcompartments (MCPs) are widespread protein-based organelles that play important roles in the global carbon cycle and in the physiology of diverse bacteria, including a number of pathogens. MCPs consist of metabolic enzymes encapsulated within a protein shell. The main roles of MCPs are to concentrate enzymes together with their substrates (to increase reaction rates) and to sequester harmful metabolic intermediates. Prior studies indicate that MCPs have a selectively permeable protein shell, but the mechanisms that allow selective transport across the shell are not fully understood. Here we examine transport across the shell of the choline utilization (Cut) MCP of *Escherichia coli* 536, which has not been studied before. The shell of the Cut MCP is unusual in consisting of one pentameric and four hexameric bacterial microcompartment (BMC) domain proteins. It lacks trimeric shell proteins, which are thought to be required for the transport of larger substrates and enzymatic cofactors. In addition, its four hexameric BMC domain proteins are very similar in amino acid sequence. This raises questions about how the Cut MCP mediates the selective transport of the substrate, products and cofactors of choline metabolism. In this report, site-directed mutagenesis is used to modify the central pores (the main transport channels) of all four Cut BMC hexamers to assess their transport roles. Our findings indicate that a single shell protein, CmcB, plays the major role in choline transport across the shell of the Cut MCP and that the electrostatic properties of the CmcB pore also impact choline transport. The implications of these findings with regard to the higher-order structure of MCPs are discussed.

## Introduction

Hundreds of bacterial species produce proteinaceous organelles known as bacterial microcompartments (MCPs) [1–5]. MCPs are polyhedral in shape, 100–150 nm in diameter and are built from thousands of protein subunits of 10 to 20 different types [6–8]. Overall, MCPs consist of metabolic enzymes encapsulated within a protein shell. The shell is used to concentrate enzymes together with their substrates (to increase reactions rates), and to sequester toxic metabolic intermediates to protect cell components. MCPs are involved in 10 or more metabolic processes ranging from carbon dioxide fixation to the catabolism of 1,2-propanediol, ethanolamine, choline, glycerol, rhamnose, fucose, fucoidan and aminoacetone [9–18]. Accordingly, MCP metabolism is important to the global carbon cycle and to the ecology of many bacteria, including enteric pathogens such as *Salmonella *, *Listeria* and certain *Escherichia coli* species.

The distinguishing feature of MCPs is a selectively permeable protein shell [19]. This shell serves to restrict the outward diffusion of metabolic intermediates that are harmful or volatile. In the case of the carboxysome, the shell impedes the outward diffusion of CO_2_, concentrating it with Rubisco and boosting reaction rates [20, 21]. In other MCPs, the shell is used to sequester toxic intermediates, especially short-chain aldehydes, thus preventing damage to DNA and other cellular components [22–25]. However, the protein shells of MCPs must also allow the entry of MCP substrates and cofactors, as well as the exit of products into central pathways to aid cell growth [19]. Hence, a key question about MCPs are the mechanisms that allow their protein-based shells to be selectively permeable to specific substrates, products, intermediates and cofactors.

Structural studies have provided information vital to understanding the selective permeability of MCP shells [7, 26]. The shells of MCPs are primarily composed of proteins having Pfam domains 03319 or 00936. Proteins with domain 03319 form pentamers that comprise the vertices of MCPs [27]. Members of this group are structurally important but are not known to mediate molecular transport across the shell [28]. Proteins having Pfam domain 00936 are a diverse family of small proteins referred to as bacterial microcompartment (BMC) domain proteins [7]. BMC proteins form relatively flat, hexameric and pseudohexameric trimers that tile side-by-side to form the facets of MCP shells. Structural studies showed that BMC hexamers have central pores whose small size (4–7 Å) and electrostatic properties suggest a role in the selective diffusion of small molecules (such as MCP substrates and products) across the MCP shell [7, 26, 29]. Site-directed mutagenesis that modified BMC hexamers supported structural studies and it is generally thought that the properties of the central pores of BMC hexamers are crucial to the selective permeability of MCP shells [19, 30, 31]. In addition, bioinformatic studies have shown that many MCP operons include multiple paralogues of BMC hexamers with varied pore chemistries and it has been proposed that these variations allow the selective diffusion of the varied metabolites associated with different types of MCPs [1, 2, 4]. A second major class of MCP shell proteins are trimers where each protomer is composed of two fused permuted BMC domains. In several instances, this class of BMC domain protein has been crystalized in two conformations: one with a large open central pore and another where the central region of the protein is closed [32, 33]. This suggests that some trimeric BMC domain proteins might act as allosteric gates that regulate the transport of larger substrates and enzymatic cofactors in response to the signal [34]. Additionally, this type of BMC trimer has been found in a stacked conformation that creates a central cavity suggesting molecular transport by an ‘airlock’ mechanism [32, 35, 36]. However, a role for permuted BMC trimers in MCP transport has not been confirmed by biochemical or genetic studies. A third major class of MCP shell proteins is trimers where each protomer has two canonical BMC domains rather than two permuted BMC domains [29]. This group is thought to have specialized functions but specifics have not been worked out.

**Fig. 1.**
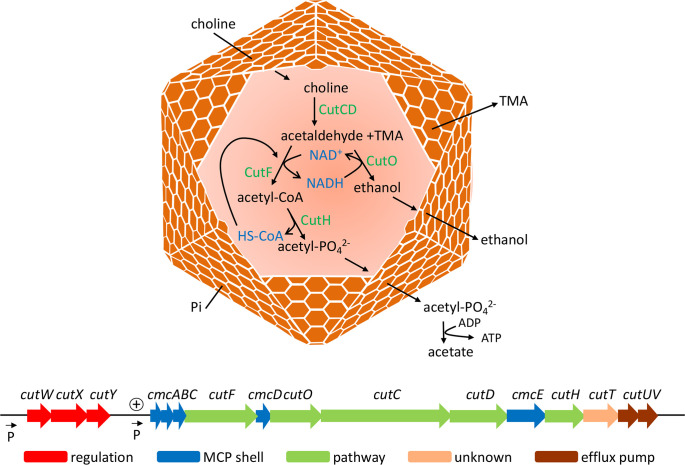
Choline utilization in *EC*536. Top: model for the Cut MCP. Choline crosses the protein shell and enters the lumen of the MCP. Choline is metabolized to acetyl-phosphate and ethanol, which exit the MCP. In the cell cytoplasm, acetyl-phosphate is used to make ATP for growth. Ethanol is excreted outside the cell. The fate of trimethylamine (TMA) has not been determined. Inorganic phosphate, which is required by CutH enters by an unknown mechanism. CutD requires a catalytic amount of S-adenyosylmethioinine but how it is acquired is unknown. (CutCD:choline TMA lyase). (CutF:acetaldehyde dehydrogenase). (CutO:alcohol dehydrogenase). (CutH:phosphotransacetylase). Bottom: the 16-gene *cut* locus. Corresponding protein accession number, left to right: WP_000926369.1, WP_001270145.1, WP_001217008.1, WP_000502008.1, WP_000502010.1, WP_001206281.1, WP_000570989.1, WP_000599365.1, WP_001288714.1, WP_000035052.1, WP_001275637.1, WP_001086628.1, WP_000564322.1, WP_000139438.1, WP_000095890.1, WP_000481835.1.

In *Escherichia coli* 536 (*EC*536), a bacterial MCP mediates choline utilization (Cut) [37]. The current model for the Cut MCP proposes that choline crosses the shell and enters the lumen where it is converted to acetaldehyde and trimethylamine (TMA) by choline-TMA lyase (Fig. 1). The acetaldehyde is further metabolized to acetyl-phosphate and ethanol, which subsequently exit the MCP into the cell cytoplasm by an unknown mechanism. Ethanol is excreted outside the cell and acetyl-phosphate is used to generate ATP for growth. The function of the Cut MCP is to sequester acetaldehyde to prevent damage to cell components and/or loss to the environment since acetaldehyde is not well-retained by lipid bilayer membranes. Based on gene clustering, it is thought that the shell of the Cut MCP of *EC*536 is composed of four BMC hexamers and one pentamer, which is typical of class II Cut MCPs (Fig. 1) [37, 38]. Three of the four BMC hexamers encoded by the *cut* locus have identical amino acid sequences in the pore and, surprisingly, the *cut* locus does not encode a BMC trimer. Current models do not explain how four hexameric BMC proteins with identical pore sequences could selectively transport the enzyme substrates, cofactors and products of choline metabolism. In this report, the four Cut BMC shell proteins and their roles in molecular transport are examined using structure-guided mutagenesis to alter their central pore regions and physiological studies. Our results indicate that one shell protein, CmcB, is primarily responsible for choline transport and the other nearly identical shell proteins have little or no role in this process. This suggests that the context of shell proteins within the higher-order structure of the Cut MCP modifies their transport capabilities.

## Results

### Genomic analysis of *EC*536 BMC domain proteins

Bioinformatic and genetic studies indicate that many MCPs are encoded by genes that cluster at a single locus, although in some cases MCP genes are dispersed among multiple chromosomal loci [2]. The *EC*536 *cut* locus includes 16 genes used for the formation of an MCP that mediates choline degradation (Fig. 1) [37]. This locus encodes only five shell proteins: one pentamer, four BMC hexamers and no trimers, which is unusual. To investigate the possibility that additional Cut MCP shell proteins might be located outside the main *cut* locus, we used the blast programme to search for proteins with recognizable amino acid sequence similarity within the *EC*536 genome. Using both canonical and permuted BMC domains as queries, BMC homologues were found at two loci: the *cut* locus, and the ethanolamine utilization (*eut*) locus. The *eut* locus encodes an MCP used for the B_12_-dependent degradation of ethanolamine [16]. Further studies using a *eut::lacZ* reporter gene showed that transcription of a *eut* locus was too low to be measured under conditions of choline metabolism whereas under conditions of ethanolamine breakdown the *eut::lacZ* reporter was expressed at 37±2 Miller units. Since *eut* genes are off or minimally expressed under conditions of choline metabolism, it is very unlikely that *eut* shell proteins would normally incorporate into the Cut MCP. It has also been shown that co-expression shell proteins for different MCPs interferes with MCP function [39]. Furthermore, we looked at choline degradation in a strain having a precise deletion of the entire *eut* locus including all the *eut* MCP shell genes. Comparing this deletion to wild-type *EC*536, we found no difference in growth on choline or in acetaldehyde production, supporting the idea that *eut* shell proteins have no role in choline metabolism (Fig. S1, available in the online version of this article). (Later in this report we show that acetaldehyde production is altered by mutations that substantially change the structure of the Cut MCP.). We conclude that for *EC*536, BMC domain proteins encoded outside the *cut* locus are not used in choline degradation and that no trimeric BMC domain proteins are required for choline metabolism.

### Construction of site-directed mutants that altered the central pores of Cut MCP shell proteins

Prior studies used site-direct mutagenesis to study the roles of the central pores of BMC domain shell proteins in the transport of 1,2-propanediol across the shell of the 1,2-PD utilization (Pdu) MCP of *Salmonella* [31]. The PduA hexamer (a major shell protein of the Pdu MCP) has six serine residues (one from each protomer) that come together to form the constriction point of its central pore [29, 40]. Mutagenesis that altered this serine showed that the size and chemical properties of the PduA central pore are critical to facilitating selective diffusion of its specific substrate into the Pdu MCP [31]. Similar to the PduA shell protein, serine residues also form the constriction points of the central pores of all four BMC shell proteins of Cut MCP (CmcA, CmcB, CmcC and CmcE). The constriction point serine residues are S39 for CmcA, CmcB, and CmcC and S38 for CmcE. The pore regions of CmcA, CmcB and CmcC are all identical and each of these proteins have >81 % identity to the other two. This raises questions about the reasons for the apparent redundancy and the relative contributions of each BMC protein to substrate transport across the MCP shell.

To investigate the roles of the central pores of CmcA, CmcB and CmcC in molecular transport across the Cut MCP shell, we made triple mutants that resulted in an S39L change in CmcA, CmcB and CmcC shell proteins. S39L variants were selected because prior studies demonstrated that the corresponding S40L variant in the PduA shell protein substantially impeded 1,2-propandiol transport into the Pdu MCP [31]. The triple mutants were made by recombineering (Fig. S2) [41]. A chromosomal mPheS-Gent cassette that deleted most of the *cmcABC* genes was introduced into the *EC*536 genome and subsequently replaced with synthetic DNA designed to introduce S39L mutations into *cmcA*, cmcB and *cmcC,* simultaneously. Replacement of the mPheS-Gent cassette by the mutagenic synthetic DNA was selected on plates containing 4-chloro-dl-phenylalanine (4 CP), which inhibits the growth of strains expressing the *mpheS* gene [42]. Subsequent phenotypic tests, PCR and DNA sequencing identified the mutants with the desired chromosomal changes and these mutants were used for further studies. However, we want to mention that DNA sequencing identified transformants that had S39L mutation(s) together with deletions between direct repeats found in the *cmcABC* genes (which are paralogues with high sequence identity) including the following: (1) a 299 base-pair deletion resulting from recombination between a 38 base-pair direct repeat found in cmcB and cmcC (GCCGCCAACGTTGAGTTGATTGGCTATGAAAACGTCGG); (2) a 595 base-pair deletion involving a 23 base-pair direct repeat in *cmcA* and *cmcC* (AAAGGCGACGTTGGCGCAGTGAA); (3) a 595 base-pair deletion between a 14 base-pair direct repeat found in cmcA and cmcC (ATCGAAACCAAAGG); (4) and a 299 base-pair deletion between a 20 base-pair direct repeat found in *cmcB* and *cmcC* (GCGGTGGATTCCGGTGTTGA).

Our interpretation is that the expected recombination replaced the mPheS-Gent cassette with the mutagenic synthetic DNA and a subsequent recombination event produced a deletion between direct repeats found in the *cmcABC* genes. Apparently, these deletions occurred at a relatively high frequency due to the direct DNA repeats found in the *cmcABC* paralogues and the high activity of the RED recombinase used for recombineering. Accordingly, all mutants used in this study were verified by PCR and DNA sequencing after curing the RED recombinase plasmid from the parental strain.

### CmcA S39L, CmcB S39L, CmcC S39L triple variants are impaired for choline utilization

**Fig. 2.**
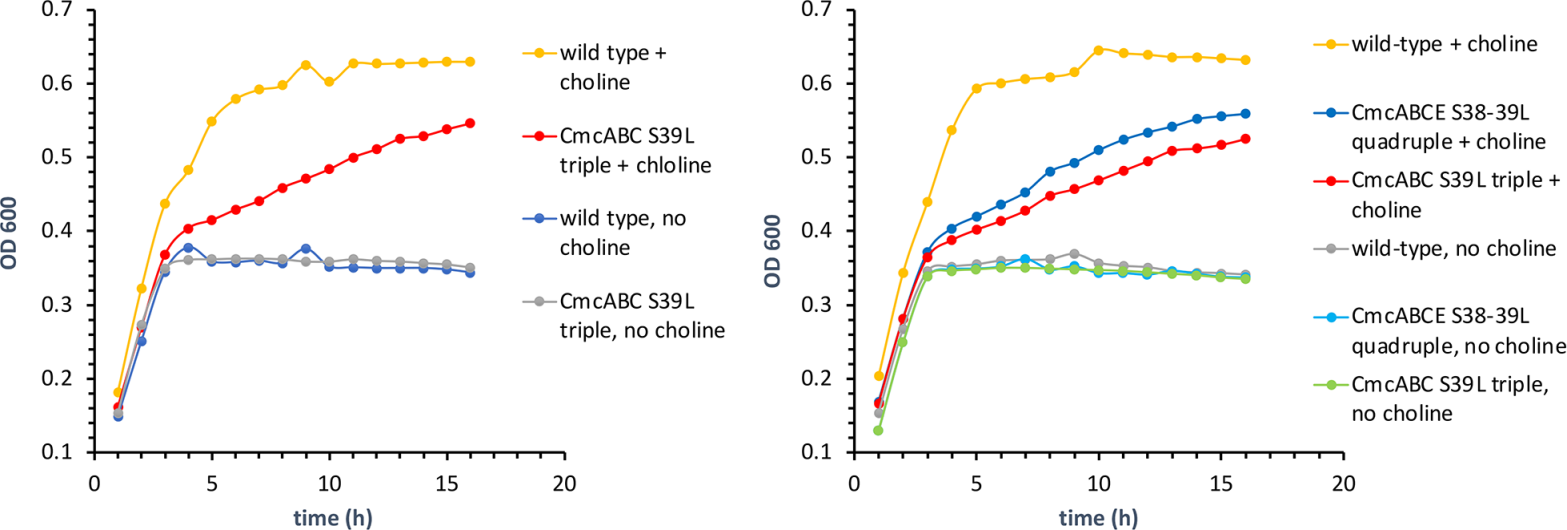
Effect of pore mutations on choline stimulation of anaerobic growth. Left panel: choline stimulation of anaerobic growth is reduced in a CmcA S39L, CmcB S39L, CmcC S39L triple variant. Right panel: a CmcE S38L mutation does not further reduce growth stimulation by choline when combined with CmcA S39L, CmcB S39L, CmcC S39L mutation. Growth curves were performed with a microplate reader.

After constructing and confirming the genotypes of the CmcA S39L, CmcB S39L, CmcC S39L triple variants, we measured their growth on complex medium with and without choline supplementation and compared their growth to wild-type *EC*536 (Fig. 2). For wild-type *EC*536, we observed choline-stimulated anaerobic growth on complex medium as previously reported [37]. Without choline, growth levelled off at OD600 of ~0.35. With choline, growth reached an OD600 of ~0.65. The triple mutant grew similarly to wild-type in the absence of choline, however, with choline, the highest OD600 value reached by the triple mutant was lower and (~0.55) and the time it took to reach this OD600 was substantially longer compared to wild-type *EC*536 most likely due to ATP limitation (Fig. 2). We also looked at the growth of a CmcA S39L, CmcB S39L, CmcC S39L, CmcE S38L quadruple variant. In this strain, the central pores of all four Cut BMC domain shell proteins were modified to impair metabolite transport. Results showed that this quadruple mutant grew similar to the CmcABC S39L triple variant; the CmcE S38L pore mutation did not further reduce choline stimulation of anaerobic growth (Fig. 2). We also looked at the CmcE S38L single pore variant and found it had no effect on choline stimulation of anaerobic growth (Fig. S3).

### CmcB is the primary shell protein involved in choline transport into the cut MCP

**Fig. 3.**
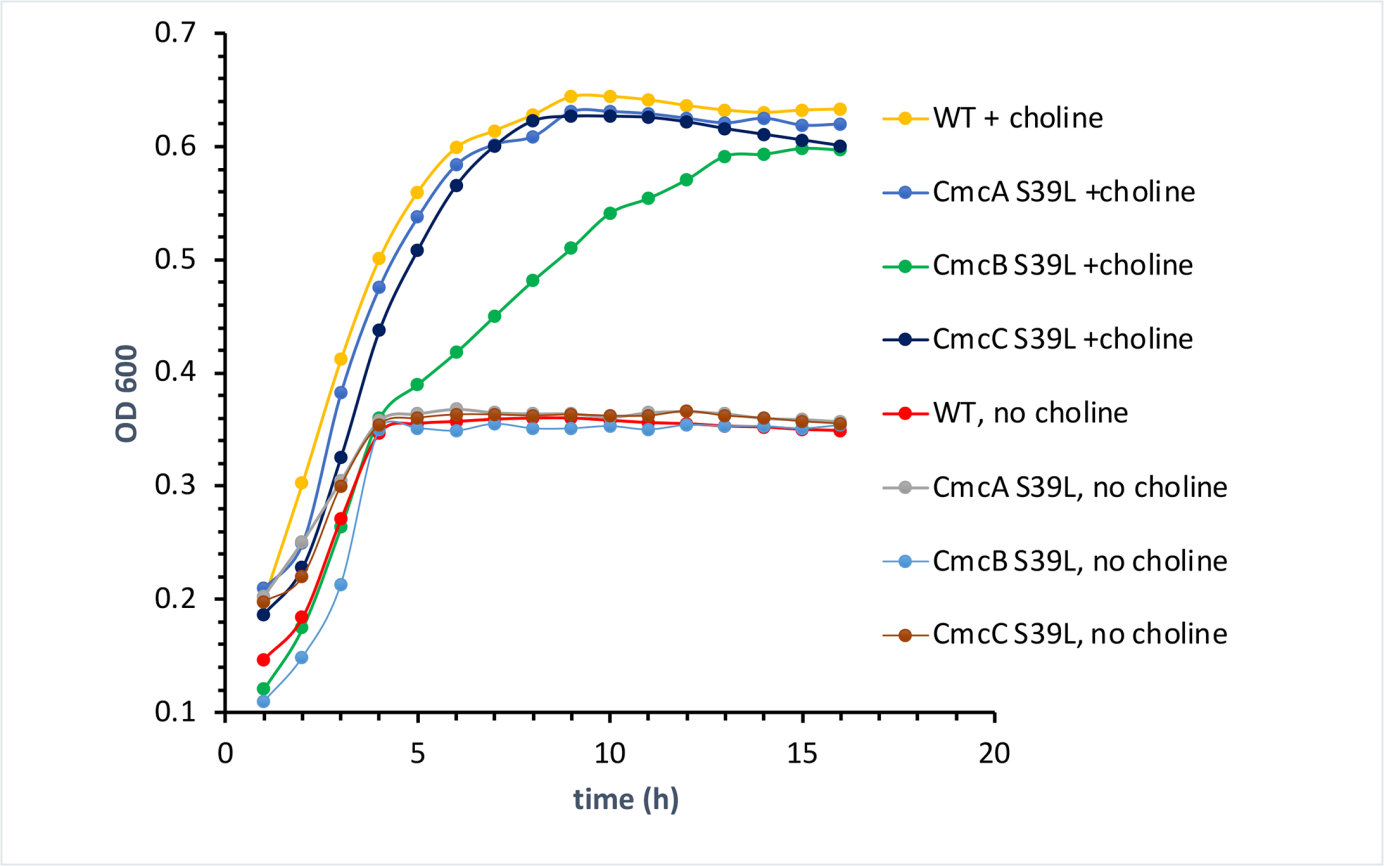
A CmcB S39L single variant impairs growth simulation by choline, but CmcA S39L, CmcC S39L or CmcE S38L single variants do not. Growth curves were done with a microplate reader.

Since the CmcABC S39L triple variant of *EC*536 showed a large reduction in choline stimulation of anaerobic growth, we next examined single and double variants to assess the relative contributions of CmcA S39L, CmcB S39L and CmcC S39L to the observed phenotype. Among the CmcA S39L, CmcB S39L and CmcC S39L single variants only CmcB S39L affected the stimulation of anaerobic growth by choline (Fig. 3). WT *EC*536 reached an OD 600 of 0.64 in the presence of choline and 0.37 in the absence of choline. Both the CmcA and CmcC single variants grew similarly to wild-type. In contrast, the CmcB S39L variant did not grow as well as WT *EC*536 anaerobically in the presence of choline. It reached an OD600 of 0.59 and approached that OD at a much slower rate. Comparing Figs 2 and 3 suggests that the CmcB S39L mutation accounts for most or all of the growth phenotype (reduced growth stimulation by choline) observed for the CmcA S39L, CmcB S39L and CmcC S39L triple variant. We also looked at all combinations of double variants among CmcA S39L, CmcB S39L and CmcC S39L. Only combinations that included the CmcB mutation showed a clear reduction in growth stimulation by choline (Fig. S4) providing further support that the CmcB S39L mutation is the primary contributor to reduced stimulation of anaerobic growth by choline.

### *cmcA*, *cmcB* or *cmcC* single deletion mutants have little effect on choline stimulation of anaerobic growth

We also looked at the effects of *cmcA*, *cmcB*, *cmcC* or *cmcE* single-deletion mutations on anaerobic growth simulation by choline in *EC*536 (Fig. S5). These individual deletions had no obvious phenotype. This is similar to results reported for other MCPs where shell gene deletions result in the formation of aberrant shells that allow the nonspecific movement of small molecules across the shell and substrate metabolism still occurs [24, 43]. Importantly, this control experiment demonstrated that the CmcB S39L pore variation was responsible for the observed phenotype rather than a general loss of function of the CmcB protein.

### Effects of CmcA, CmcB and CmcC pore mutations on acetaldehyde production during anaerobic growth of *EC*536 in the presence of choline

**Fig. 4.**
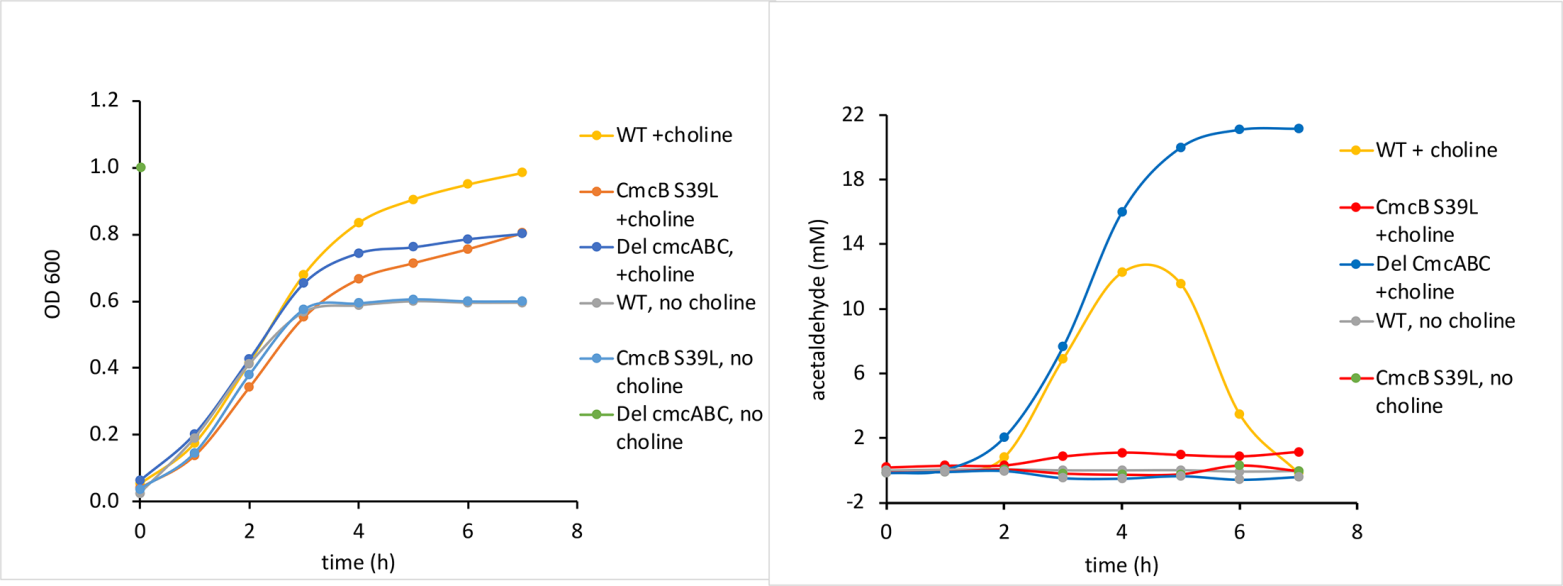
Effect of a CmcB S39L single mutation and a *cmcA*, *cmcB, cmcC* triple deletion mutation on stimulation of anaerobic growth by choline and on acetaldehyde production. Left: growth curves were performed in sealed tubes to facilitate sampling for aldehyde measurements.

For experiments where both growth and acetaldehyde were measured, cells were grown in sealed anaerobic culture tubes (to facilitate sampling for aldehyde measurements) rather than in a microplate reader as was done in the studies described above. Under these conditions, choline stimulated anaerobic growth of *EC*536, but final cell densities measured were higher than seen for growth curves done in microplates (Fig. 4). For *EC*536, the OD600 reached values of 0.99 and 0.6 with and without choline, respectively. The CmcB S39L variant showed reduced stimulation of anaerobic growth by choline reaching OD600 values of 0.8 with choline and 0.6 without choline. The CmcB S39L variant also took much longer to reach its final OD on growth medium containing choline than did the WT *EC*536. We also tested a *cmcABC* triple deletion as a control for a nonfunctional microcompartment shell. This strain grew similar to wild-type without choline, but slower than wild-type in growth medium containing choline most likely due to acetaldehyde toxicity. These results are similar to those seen for other MCPs where disruption of the shell does not impair MCP metabolism but leads to the build-up of toxic aldehydes that slow cell growth and increase DNA damage [24].

We also measured the acetaldehyde present in the growth medium over time using the same cultures for which growth was determined (Fig. 4). The *cmcABC* triple deletion mutant produced the highest maximum amount of acetaldehyde (21 mM). WT *EC*536 produced a maximum of 12 mM acetaldehyde (at *t*=4 h). Notably, the CmcB S39L variant produced considerably less acetaldehyde reaching a maximum of approximately 1 mM. The high levels of aldehyde produced by the *cmcABC* triple deletion are likely due to an improperly formed Cut MCP that is no longer selectively permeable to small molecules. Prior studies showed that mutations that break the Pdu MCP result in production of higher amounts of propionaldehyde (~20 mM) thereby inhibiting cell growth and increasing DNA damage [24]. Hence, the high acetaldehyde levels produced by the *cmcABC* triple deletion mutant likely account for the reduction in choline simulated growth. We also found that WT *EC*536 produced approximately 12 mM acetaldehyde during anaerobic growth with choline supplementation. This is higher compared to propionaldehyde production by wild-type *Salmonella* during MCP-dependent growth on 1,2-propanediol (~2 mM) [24]. The aldehyde levels reached are likely determined by the specific organism and growth conditions used. The most notable finding was that the acetaldehyde levels produced by the CmcB S39L variant were much lower than was observed for WT *EC*536 (1 mM compared to 12 mM). This indicates the rate at which the choline TMA lyase converts choline to acetaldehyde and TMA is slower in the mutant than the WT since this reaction is the sole source of acetaldehyde production during choline degradation (Fig. 1).

### Electrostatics

**Fig. 5.**
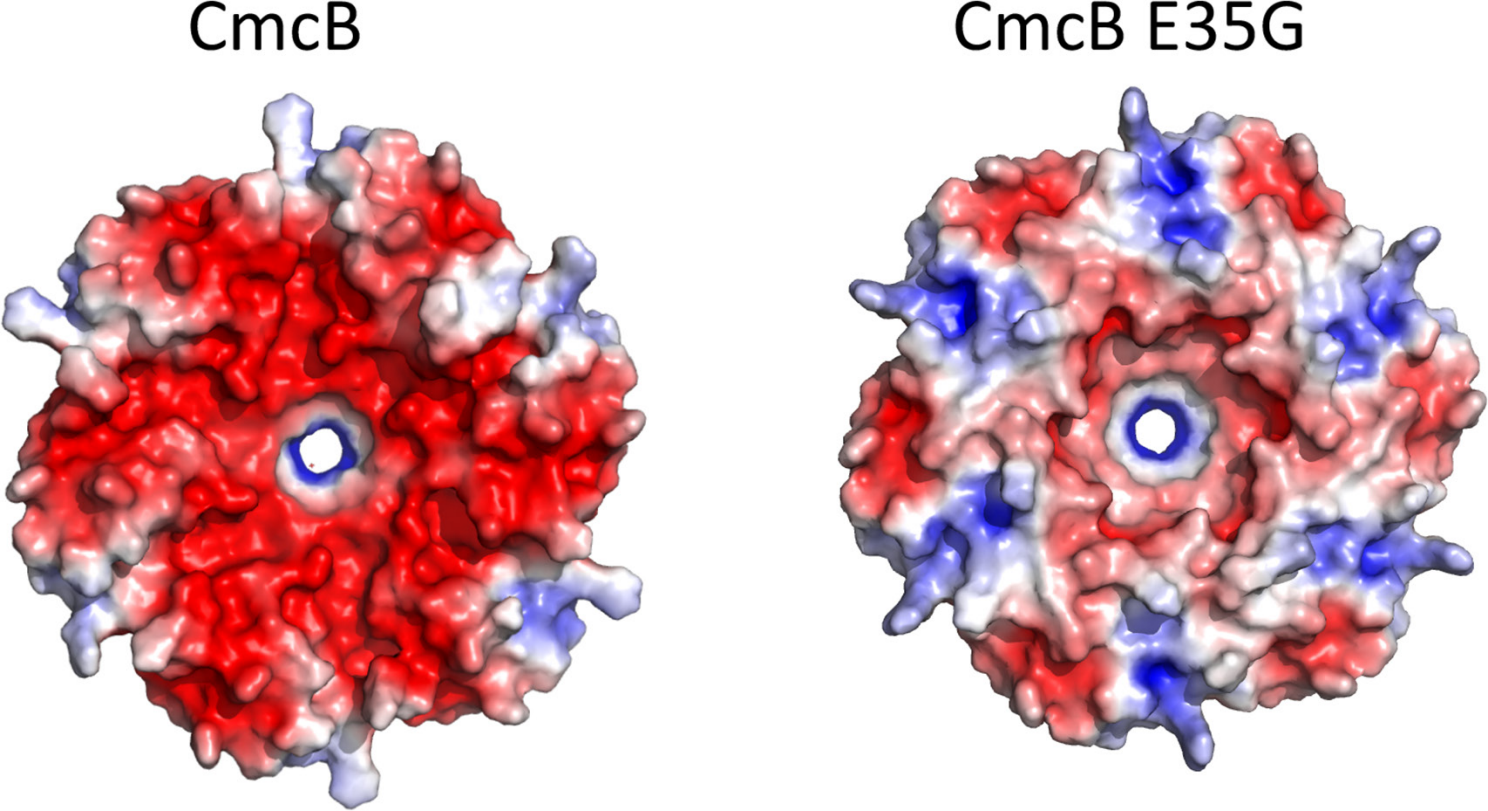
Electrostatic maps of the concave surfaces of CmcB and CmcB E35G. Maps were made with the PyMol APBS plugin using previously published crystal structures CmcB and CmcB E35G (7mpw and 7mn4) and default dielectric values.

**Fig. 6.**
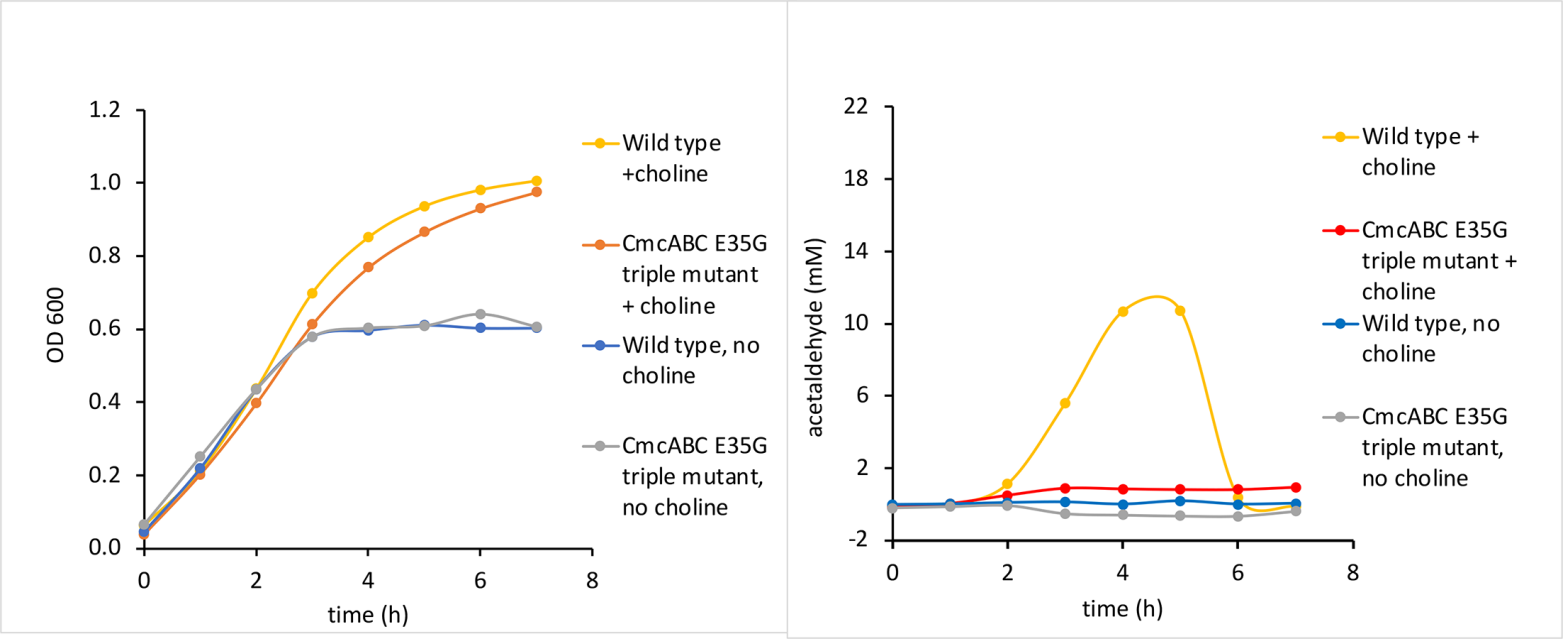
Effects of a CmcA E35G, CmcB E35G, CmcC E35G triple variant on choline stimulation of anaerobic growth and acetaldehyde production. Cells were grown in sealed tubes for these studies.

**Fig. 7.**
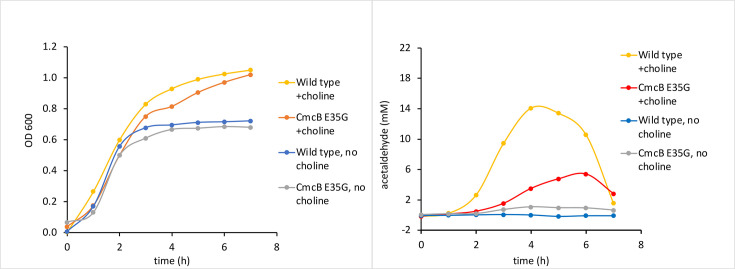
Effects of CmcB E35G single variant on choline stimulation of anaerobic growth and acetaldehyde production. Cells were grown in sealed tubes for these studies.

Several groups have proposed that the electrostatic properties of the pore regions of BMC shell proteins might play a role in the selective diffusion of small molecules across MCP shell [19]. The choline MCP appears particularly well-suited to test this hypothesis since its substrate (choline) is a quaternary amine that carries a fixed positive charge. Two residues that affect the electrostatics around the pore region of the CmcA, CmcB and CmcC shell proteins are residues K11 and E35. K11 is on the flat surface and E35 is on the concave surface (Fig. 5). We tested the effects of the *EC*536, CmcA E35G, CmcB E35G, CmcC E35G triple variant on choline stimulation of anaerobic growth and aldehyde production (Fig. 6). Growth stimulation was similar for wild-type and the mutant. However, levels of acetaldehyde present in growth medium were substantially reduced for the mutant compared to wild-type. Similar results were seen for the CmcB E35G single variant suggesting that changing the electrostatics of the CmcB pore had the major influence on the reduction of acetaldehyde levels seen for the triple mutant (Fig. 7). Lower acetaldehyde levels indicate that the activity of choline TMA lyase was reduced suggesting impaired choline transport. We also note that the CmcB E35G single variant produced more acetaldehyde (5 mM) than the CmcABC E35G triple variant (1 mM) suggesting that the negative charges on the CmcA and CmcC proteins might also have some effect on choline transport. In other experiments, we showed that the CmcABC K11G triple variant had little effect on anaerobic growth stimulation by choline or acetaldehyde production (Fig. S6).

### Electron microscopy

**Fig. 8.**
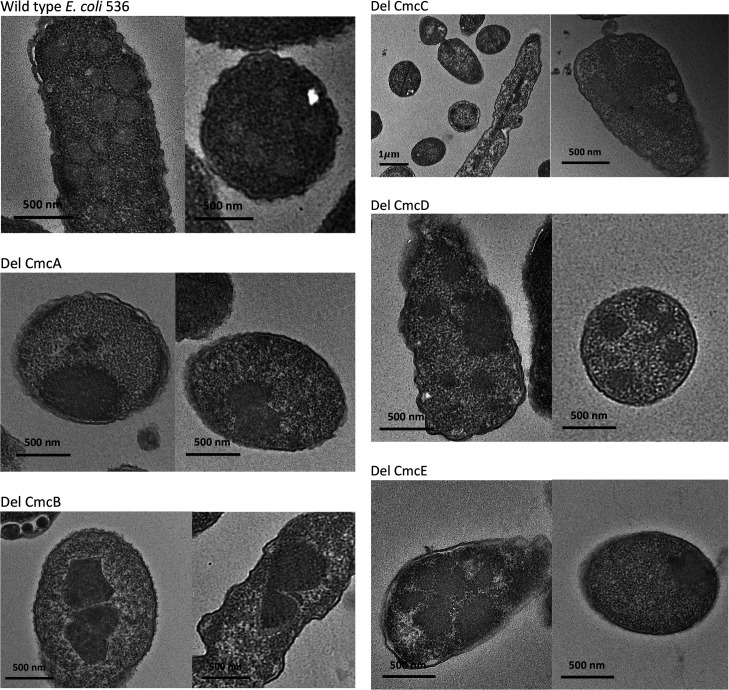
Electron microscopy of MCPs formed by wild-type *EC*536 and shell gene deletions. Precise individual deletion mutations of each shell gene were analysed using thin sections. Cells were grown anaerobically in the presence of choline. No visible MCPs were form without added choline.

Each of the *cmcABCDE* shell genes were deleted individually and the effects of these deletions on MCP formation were evaluated by EM thin sections (Fig. 8). These genes encode four BMC domain shell proteins (CmcA, CmcB, CmcC and CmcE) as well as one shell pentamer (CmcD). Wild-type *EC*536 almost exclusively produced MCPs that were roughly polyhedral ranging in size from about 100–200 nm. The *cmcA* deletion mutant produced many large amorphous protein aggregates and polar bodies that were not present in the wild-type. Cells with a *cmcB* deletion frequently produced large bodies surrounded by a shell with sharp corners. The *cmcC* deletion mutant produced long thick rods (or tubes) that in many instances tethered dividing cells together as well as a few thin rods. The *cmcD* deletion (pentamer) produced many apparently normal looking MCPs and many large protein aggregates. Strains with a *cmcE* deletion, produced protein aggregates of varied sizes, but most cells lacked large proteinaceous structures or MCPs altogether.

## Discussion

The studies reported here focused on the role of the CmcABCE BMC domain proteins in molecular transport across the shell of the Cut MCP. Results indicated that the hexameric CmcB shell protein plays a major role in the transport of choline into the Cut MCP. A mutation (S39L) that blocked the pore in CmcB caused a reduced level of choline stimulated anaerobic growth and also produced lower levels of acetaldehyde compared to wild-type *EC*536. Both of these phenotypes are consistent with slower uptake of choline into the Cut MCP where it is converted to acetaldehyde, which is further metabolized to stimulate cell growth (Fig. 1). In addition, the S39L variant is analogous to the PduA S40L variant, which restricts its central pore impeding uptake of 1,2-propanediol into the Pdu MCP reducing growth of *Salmonella* on 1,2-propanediol [31]. We also showed that a *cmcB* single deletion mutant did not affect choline stimulation of anerobic growth showing that this phenotype was specific to the S39L mutation and not due to inactivation of CmcB. In the deletion, choline likely enters in a nonspecific manner due to abnormal formation of the shell as was seen in prior studies on the Pdu MCP [24], and which is supported by the electron microscopy reported here (Fig. 8). Hence, overall, the results indicate that CmcB has an important role in the transport of choline into the Cut MCP.

In contrast, CmcA, CmcC and CmcE had no obvious role in choline transport into the Cut MCP. CmcA S39L, CmcC S39L and CmcE S38L single mutations had little effect on choline stimulated growth or acetaldehyde production. This is somewhat surprising since CmcA and CmcC are >81 % identical to CmcB and are expected to have identical pore structures. A possible explanation is that CmcA and CmcC interact with other MCP components in such a way that prevents them from participating in choline transport. Associations with MCP lumen enzymes might block their central pores or they might stack face-to-face forming dodecamers (as *in vitro* studies have suggested for other BMC proteins) in a way that alters the configuration of their pores and potentially their transport abilities [44]. Alternatively, CmcB might interact with choline TMA lyase to facilitate transport. Overall, findings suggest that CmcB plays a central role in choline transport into the Cut MCP, but that CmcA, CmcC and CmcE have little effect on this process perhaps due to specific interactions with other Cut MCP components.

We also used site-directed mutagenesis to change the overall charge around the pores of the CmcABC shell proteins. We looked at the effects of CmcABC K11G and E35G triple variants on growth simulation by choline and acetaldehyde production. A CmcABC E35G triple variant showed reduced aldehyde production, but growth stimulation by choline was similar to wild-type. Our interpretation is that the E35G change reduced choline uptake into the Cut MCP, reducing the rate at which choline was converted to acetaldehyde, but that this reduction was not sufficient to reduce anaerobic growth stimulation. Further tests showed that most of the reduction in aldehyde production seen in the CmcABC E35G triple variant was due to the CmcB E35G mutation, providing further support of a major role for CmcB in choline transport into the Cut MCP. Presumably, the negative charge around the pore of CmcB enhances choline uptake though attractive forces between choline and the concave side of the pore (Fig. 5). In contrast, the CmcABC K11G triple variant had no obvious effect on growth stimulation by choline or acetaldehyde production so the positive charge around the flat face of the CmcABC pores that arises from the K11 residue appears to have a minimal role in choline transport.

We also presented an EM study of *EC*536 strains using precise single deletions of the *cmcA*, *cmcB*, *cmcC*, *cmcD* or cmcE genes (Fig. 8). Each individual deletion had a large effect on MCP structure suggesting that each individual shell protein has a substantial structural role. In the case of the Pdu MCP (one of the best-studied MCPs) mutations that result in the formation of aberrant MCPs allow nonspecific movement of small molecules across the shell and the substrate is still metabolized, but with the accumulation of propionaldehyde to toxic levels [24, 43]. The situation appears analogous for the Cut MCP. Strains with a *cmcA*, *cmcB* or *cmcC* deletion still metabolize choline but the aberrant MCP formed are likely to cause acetaldehyde to accumulate to higher levels as shown for the *cmcABC* triple deletion mutant (Fig. 4). EM studies also showed that individual deletions of *cmcA*, *cmcB*, *cmcC*, *cmcD* or *cmcE* genes produced characteristic structural phenotypes suggesting each shell protein has a specific structural role and a specific set of interactions with other MCP components despite their high sequence similarity.

The Cut MCP is unusual in lacking trimeric BMC domain proteins. It has been proposed that trimers with BMC domains are used to transport larger molecules including enzymatic cofactors across MCP shells [19]. However, it has also been shown that MCP cofactors can be internally recycled since MCPs encapsulate enzymes that both form and consume NAD^+^ and HS-CoA (Fig. 1) [45, 46]. The Cut MCP mediates a strictly anaerobic process. Hence, internal cofactor recycling might play a primary role and trimeric BMC domain proteins might be unnecessary. In contrast, other catabolic MCPs support aerobic metabolism where NADH generated internally within the MCP must be oxidized by the electron transport chain if cells are to take advantage of the ATP gains possible from electron transport phosphorylation and this would likely require cofactor transport across the shell.

## Methods

### Chemicals and reagents

Antibiotics were from Sigma Chemical Company (St. Louis, MO). Choice *Taq* Blue Master Mix was from Denville scientific (Holliston, MA). *KOD* Hot Start Master Mix was from EMD Millipore (Billerica, MA). Restriction enzymes and HiFi DNA Assembly Master Mix were from New England Biolabs (Beverly, MA). Antibiotics, choline chloride, 4-chloro-dl-phenylalanine and other chemicals were from Fisher Scientific (Pittsburgh, PA).

### Bacterial strains and growth conditions

The bacterial strains used in this study are listed in Table 1. BSL2 precautions were used for all growth studies. The rich medium used was lysogeny broth (LB), also known as Luria–Bertani medium (Becton, Dickinson and Company, Franklin Lakes, NJ) [47]. MacConkey-choline indicator plates were prepared using Difco MacConkey Agar Base supplemented with 0.8 % choline chloride from a 50 % stock solution of choline chloride that was adjusted to pH 7.0.

**Table 1.** Strains used in this study*

Strain	Genotype	Source
BE2278	wild-type *E. coli* 536	Gift from Harry L. T. Mobley
BE2288	/pKD46	lab collection
BE2841	*cmcABC*::mpheS-Gent	this study
BE2848	*cmcABC*::mpheS-Gent/pKD46	this study
BE2879	CmcA S39L, CmcB S39L CmcC S39L	this study
BE2904	CmcE S38L	this study
BE2906	CmcA S39L CmcB S39L CmcC S39L CmcE S38L	this study
BE3137	CmcA S39L	this study
BE3060	CmcB S39L	this study
BE3139	CmcC S39L	this study
BE2928	CmcB S39L CmcC S39L	this study
BE2929	CmcA S39L CmcC S39L	this study
BE2948	CmcA S39L CmcB S39L	this study
BE2354	Δ*cmcA*::frt	lab collection
BE2606	Δ*cmcB*::frt	lab collection
BE2308	Δ*cmcC*::frt	lab collection
BE2350	ΔcmcD::frt	lab collection
BE2352	Δ*cmcE*::frt	lab collection
BE2878	Δ*cmcABC*	this study
BE3145	CmcA E35G CmcB E35G CmcC E35G	this study
BE3062	CmcB E35G	this study
BE3142	CmcA K11G CmcB K11G CmcC K11G	this study
BE2707	Δ*lacZ*	this study
BE2802	Δ*lacZ*/pKD46	this study
BE2917	Δ*lacZ eutR-hemF* intergenic region::mPheS-Gent	this study
BE2918	Δ*lacZ, eutR-hemF* intergenic region::mPheS-Gent/pKD46	this study
BE2836	Δ*lacZ eutR-hemF* intergenic region::*lacZ*	this study
BE3165	Δ*lacZ* Δeut operon	this study

*The strains used in the study are derivative of *E. coli* 536.

### Growth curves and acetaldehyde assays

Anaerobic growth curves were performed in both microplates and in sealed tubes as described using 71 mM choline choride or as indicated in the test [37]. Acetaldehyde assays were preformed using 3-methyl-2-benzothiazolinone-hydrazonehydrochloride (MBTH) in a 96-well microplate format. Culture media of cells growing in a sealed tube were sampled by syringe (10–40 µl). The sample was diluted as appropriate and added to a microplate well that contained 286 µl, 100 mM K+citrate buffer pH 3.6, 143 µl 0.1 % MBTH, and 286 µl double deionized water (less the sample volume) and incubated for 15 min at 37 °C. Then, 285 µl of double deionized water was added that the absorbance at 305 nm was determined. Quantitation was done by comparison to a standard curve where the linear range was 0.01–0.2 mM (final concentration in the assay) with ε306=13,300 mM^−1^ cm^−1^. Controls showed that the growth medium did not influence the assay results.

### Electron microscopy and β-galactosidase assay

Imbedding, sectioning and electron microscopy were carried out as described [48]. β-galactosidase assays were also performed as described [39].

### Molecular biology methods

Agarose gel electrophoresis, plasmid purification, PCR, restriction digests, ligation reactions and electroporation were carried out using standard protocols as described [15, 49]. Choice *Taq* Blue Master Mix was used for colony PCR. *KOD* DNA polymerase was used for amplification of plasmid templates. Plasmid DNA was purified using Qiagen products (Qiagen, Chatsworth, CA) according to the manufacturer’s instructions. Following restriction digestion or PCR amplification, DNA was purified using Monarch DNA purification and gel extraction kits (New England Biolabs). For ligation of DNA fragments, NEBuilder HiFi DNA Assembly Master Mix was used according to the manufacturer’s instructions (New England Biolabs). Transformation was done by electroporation with a Bio-Rad gene pulser Xcell and the preprogramed settings for *E. coli *.

### Recombineering

Recombineering was carried out by linear transformation of PCR products using pKD46 for expression of the lambda RED recombinase [41]. For construction of *cmcABC* triple mutants, primers cmc-F (5′-GCAGCGGATGCGATGTGTAAATCCGCCAACGTTGAACTGATTGGCGAATTCGCGGCCGCTTCTAG-3′) and cmc-R (5′-CGCGGCATTCACTGCGCCAACGTCGCCTTTTACCATCGCGGTGACCGTTATTAGGTGGCGGTACTT-3′ were used were used to amplify the mPheS-Gent cassette of pMG1. These primers include 50 base-pair flanking sequences for replacement of bases 100–718 of the *EC*536 *cmcABC* genes with the mPheS-Gent cassette. After amplification, about 200 ng of DNA was introduced into strain BE2288 (*EC*536/pKD46) by electroporation. Transformants were selected on tryptone yeast extract gentamycin (20 µg ml^−1^) plates and screened for the correct insertion site by colony PCR with primers PJ1-FF (5′-CCACTCCAGCACAAATACATAAATC-3′) and UNK-FR (5′-TTTGCCAGTGCTTCAGCG-3′). Amplified DNA of the correct size was purified using a Monarch DNA purification kit (New Englands Biolabs) and sequenced to verify the chromosomal location of the mPheS-Gent cassette. About 200 ng of mutagenic DNA obtained by PCR amplification of synthetic DNA obtained from Genscript (Piscataway, NJ) was introduced into strain BE2848 (*EC*536 *cmcABC*::mpheS-Gent/pKD46) by electroporation. Replacement of the mPheS-Gent cassette was selected on yeast extract glucose plates supplemented with 16 mM DL-4-chlorophenylalanine (inhibits strains expressing the *mpheS* gene). 4 CP-resistant transformants were single colony purified on LB at 37 °C and screened for gentamycin resistance, choline degradation and ampicillin resistance. For strains that were both gentamycin and ampicillin sensitive (indicates replacement of the mPheS-gent cassette and loss of the lambda RED plasmid) colony PCR with primers PJ1-FF and UNK-FR was used to amplify the *cmcABC* region for DNA sequencing. Mutants with the desired DNA sequence were saved for further study. This approach allowed the construction of mutants with specific amino acid changes in the pores of three different shell proteins (CmcABC), simultaneously. An analogous approach was used to construct the other mutants used in this study by varying the primers used for amplification of the mPheS gent cassette, for colony PCR and DNA sequencing and the mutagenic DNA. A precise deletion of the *cmcABC* genes was also made by replacing the mPheS-Gent cassette of BE2848 with a 500 base-pair gBlock (IDT). This deletion removed the start codon of the *cmcA* gene and ended 22 upstream of the *cutF* gene leaving its ribosome binding site intact. To make the *eut* operon *lacZ* transcriptional reporter, we first made a precise deletion of the *lacZ* gene then inserted an mPheS-Gent cassette into the *eutR-hemF* intergenic region. Next we constructed a plasmid that carried a *lacZ* transcriptional reporter (which includes *lacZ* and needed translation signals) flanked on both sides by 500 base-pair sequences to allow replacement of the mPheS-Gent cassette. The *lacZ* reporter and flanking sequences were amplified by PCR and transformed into strain BE2918 by electroporation. DNA sequencing showed that the resulting strain had a *lacZ* transcriptional reporter inserted next to the stop codon of the *eutR* gene. Insertion of *lacZ* just downstream of the *eutR* regulatory protein is necessary for normal *eut* operon induction [50]. To make the precise deletion of the *eut* operon an mPheS-Gent cassette was inserted into the *eutR-hemF* intergenic region. The cassette was replaced with 500 bp gBlock that removed the start codon of *eutS*, the stop codon of *eutR* and all the DNA between. The intergenic region between *eutR* and *hemF* was left intact.

### Construction of pMG1 containing the mPheS-gent cassette

An mPheS-Gent cassette was synthesized as a gBlock (Integrated DNA Technologies, Coralville, IA). The cassette was cloned into plasmid pJD141, cut with *Eco*RI and *Pst*I, by Gibson Assembly using NEB Hi-Fi DNA Assembly Master Mix. The DNA sequence of the resulting plasmid (pMG1) was verified (Table S1).

## Supplementary Data

Supplementary material 1
