# Supplementary material for: A single shell protein plays a major role in choline transport across the shell of the choline utilization microcompartment of Escherichia coli 536
Source: Microbiology (Reading). 2023 Nov 16;169(11):001413. doi: 10.1099/mic.0.001413 (PMC10710832; doi:10.1099/mic.0.001413)
Supplement: Supplementary material 1 [file mic-169-1413-s001.pdf]

Supplementary

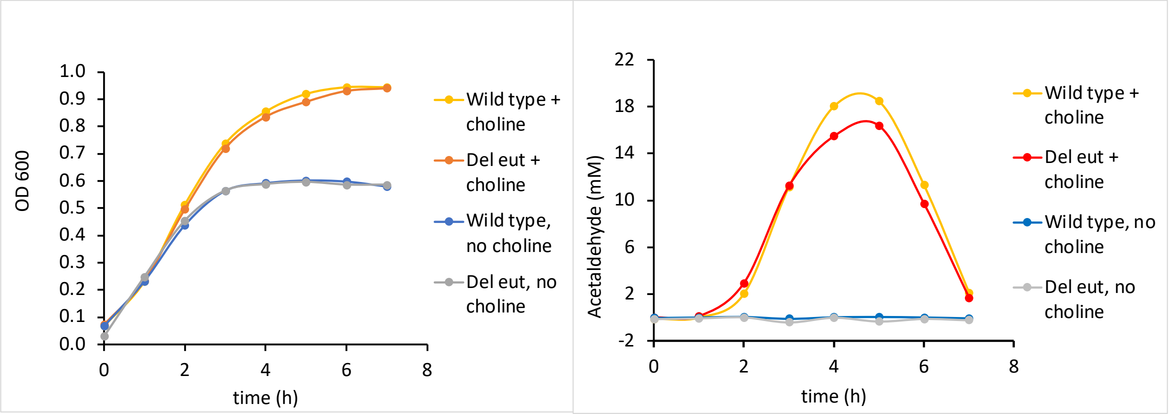

Figure S1. Effects of a *eut* locus deletion on choline degradation by *EC*536. A deletion of the entire *eut* locus of *EC*536 had no obvious effect on the stimulation of anaerobic growth by choline or on acetaldehyde production during choline metabolism. This control supports the idea the BMC domain shell proteins found in the *eut* operon have no role in choline breakdown. Cells were grown in sealed tubes for these measurements.

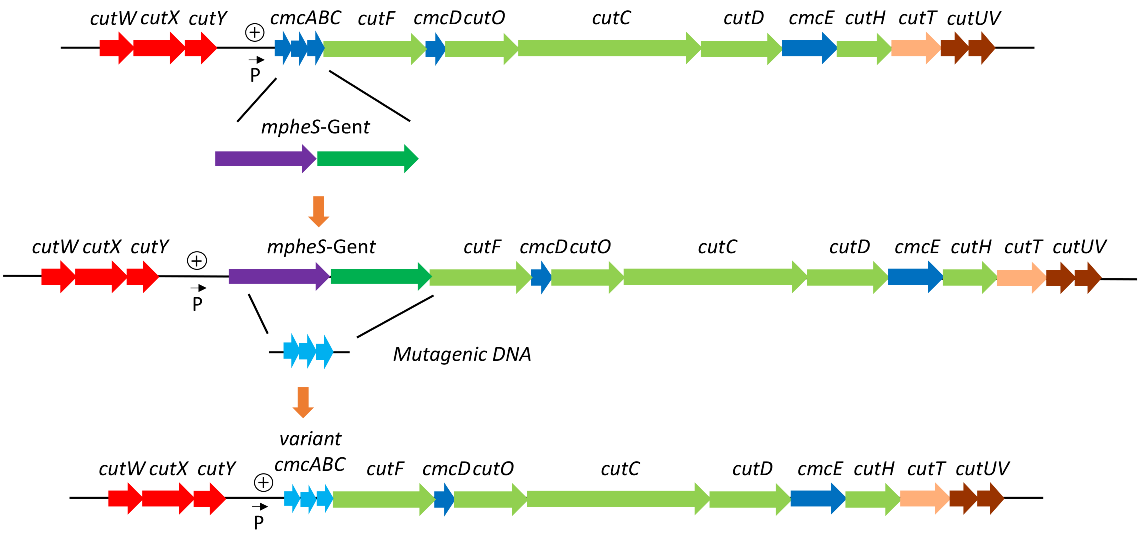

Figure S2. Diagram of recombineering approach used to construct shell gene variants. First, most of the *cmcABC* genes were replaced with an *mpheS*-Gent cassette selecting gentamycin resistance. Next, the *mpheS*-Gent cassette is replaced with mutagenic DNA selecting for 4-CP resistance. The lambda RED recombinase system was used to increase recombination frequency. The resulting mutants were verified by phenotypic testing, PCR and DNA sequencing.

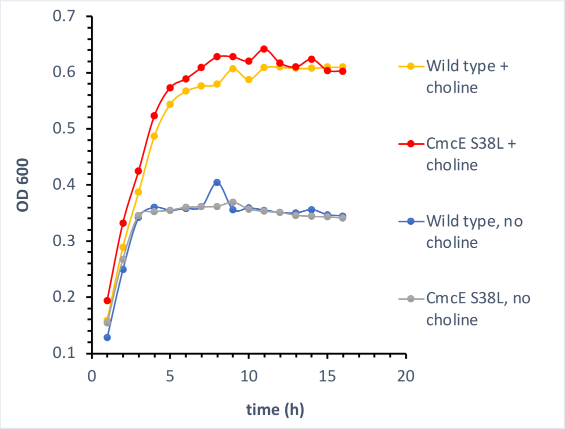

Figure S3. A CmcE S38L pore mutation did not affect the stimulation of anaerobic growth by choline. The microplate method was used to measure growth.

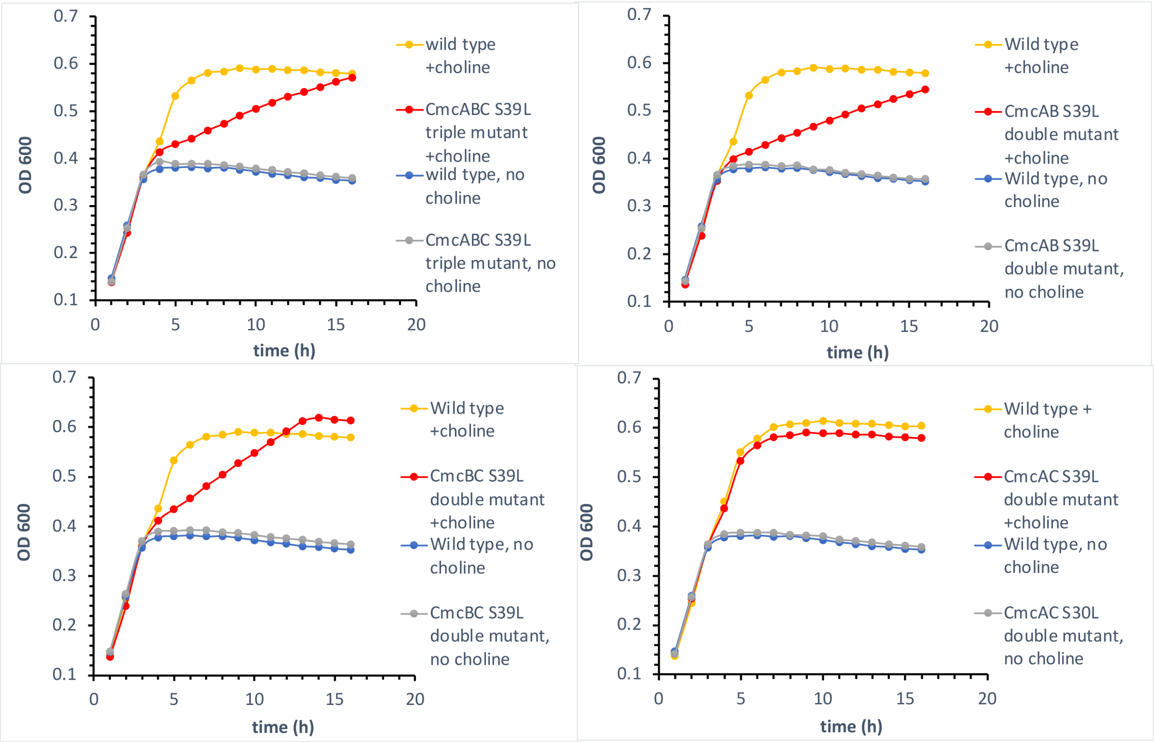

Figure S4. Anaerobic growth stimulation by choline in Cmc S39L mutants. Upper left. Choline growth stimulation in CmcA S39L, CmcB S39L, CmcC S39L triple mutant compared to wild type. Upper right. A CmcA S39L, CmcB S39L double mutant compared to wild type. Bottom left. A CmcB S39L, CmcC S39L double mutant compared to wild type. Bottom right. CmcA S39L, CmcC S39L double mutant compared to wild type. Growth curves were performed with a microplate reader.

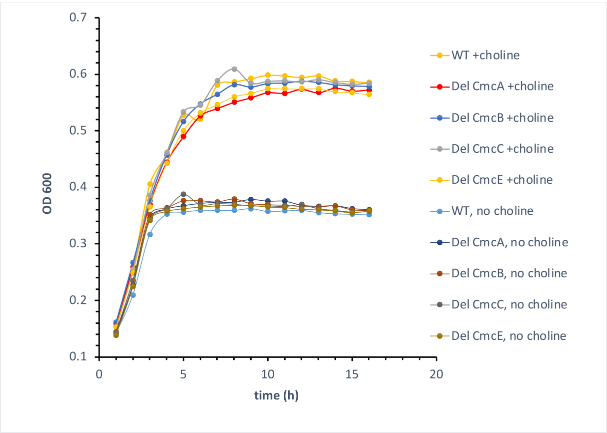

Figure S5. Precise deletions of the *cmcA*, *cmcB, cmcC* or *cmcE* genes had little effect on the stimulation of anaerobic growth by choline.

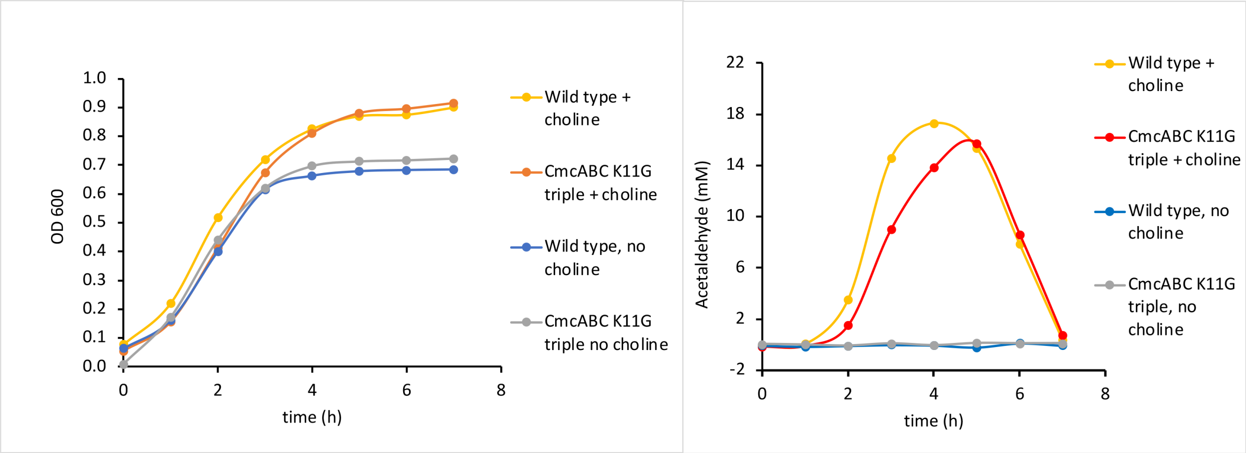

Figure S6. CmcA K11G, CmcB K11G, CmcC K11 triple mutants did not affect anaerobic growth stimulation by choline or acetaldehyde production during choline metabolism

Table S1
DNA sequence of plasmid pMG1 constructed for use in recombineering.

TGGAATTCGCGGCCGCTTCTAGAGTTGACGGCTAGCTCAGTCCTAGGTACAGTGCTAGCTACTAGAGAA
AGAGGAGAAATACTAGATGTCACATCTCGCAGAACTGGTTGCCAGTGCGAAGGCGGCCATTAGCCAGG
CGTCAGATGTTGCCGCGTTAGATAATGTGCGCGTCGAATATTTGGGTAAAAAAGGGCACTTAACCCTTC
AGATGACGACCCTGCGTGAGCTGCCGCCAGAAGAGCGTCCGGCAGCTGGTGCGGTTATCAACGAAGCG
AAAGAGCAGGTTCAGCAGGCGCTGAATGCGCGTAAAGCGGAACTGGAAAGCGCTGCACTGAATGCGC
GTCTGGCGGCGGAAACGATTGATGTCTCTCTGCCAGGTCGTCGCATTGAAAACGGCGGTCTGCATCCGG
TTACCCGTACCATCGACCGTATCGAAAGTTTCTTCGGTGAGCTTGGCTTTACCGTGGCAACCGGGCCGGA
AATCGAAGACGATTATCATAACTTCGATGCTCTGAACATTCCTGGTCACCACCCGGCGCGCGCTGACCAC
GACACTTTCTGGTTTGACACTACCCGCCTGCTGCGTACCCAGACCTCTGGCGTACAGATCCGCACCATGA
AAGCCCAGCAGCCACCGATTCGTATCATCGCGCCTGGCCGTGTTTATCGTAACGACTACGACCAGACTCA
CACGCCGATGTTCCATCAGATGGAAGGTCTGATTGTTGATACCAACATCAGCTTTACCAACCTGAAAGGC
ACGCTGCACGACTTCCTGCGTAACTTCTTTGAGGAAGATTTGCAGATTCGCTTCCGTCCTTCCTACTTCCC
GTTTACCGAACCTTCTGCGGAAGTGGACGTCATGGGTAAAAACGGTAAATGGCTGGAAGTGCTGGGCT
GCGGGATGGTGCATCCGAACGTGTTGCGTAACGTTGGCATCGACCCGGAAGTTTACTCTGGTTTCGGCT
TCGGGATGGGGATGGAGCGTCTGACTATGTTGCGTTACGGCGTCACCGACCTGCGTTCATTCTTCGAAA
ACGATCTGCGTTTCCTCAAACAGTTTAAATAACGAATCCATGTGGGAGTTTATTCTTGACACAGATATTTA
TGATATAATAACTGAGTAAGCTTAACATAAGGAGGAAAAACATATGTTACGCAGCAGCAACGATGTTAC
GCAGCAGGGCAGTCGCCCTAAAACAAAGTTAGGTGGCTCAAGTATGGGCATCATTCGCACATGTAGGCT
CGGCCCTGACCAAGTCAAATCCATGCGGGCTGCTCTTGATCTTTTCGGTCGTGAGTTCGGAGACGTAGCC
ACCTACTCCCAACATCAGCCGGACTCCGATTACCTCGGGAACTTGCTCCGTAGTAAGACATTCATCGCGC
TTGCTGCCTTCGACCAAGAAGCGGTTGTTGGCGCTCTCGCGGCTTACGTTCTGCCCAAGTTTGAGCAGCC
GCGTAGTGAGATCTATATCTATGATCTCGCAGTCTCCGGCGAGCACCGGAGGCAGGGCATTGCCACCGC
GCTCATCAATCTCCTCAAGCATGAGGCCAACGCGCTTGGTGCTTATGTGATCTACGTGCAAGCAGATTAC
GGTGACGATCCCGCAGTGGCTCTCTATACAAAGTTGGGCATACGGGAAGAAGTGATGCACTTTGATATC
GACCCAAGTACCGCCACCTAATAACGCTGATAGTGCTAGTGTAGATCGCTACTAGAGCCAGGCATCAAA
TAAAACGAAAGGCTAAGTCGAAAGACTGGGCCTTTCGTTTTATCTGTTGTTTGTCGGTGAACGCTCTCTA
CTAGAGTCACACTGGCTCACCTTCGGGTGGGCCTTTCTGCGTTTATATACTAGAAGCGGCCGCTGCAGGC
TTCCTCGCTCACTGACTCGCTGCGCTCGGTCGTTCGGCTGCGGCGAGCGGTATCAGCTCACTCAAAGGC
GGTAATACGGTTATCCACAGAATCAGGGGATAACGCAGGAAAGAACATGTGAGCAAAAGGCCAGCAAA
AGGCCAGGAACCGTAAAAAGGCCGCGTTGCTGGCGTTTTTCCATAGGCTCCGCCCCCCTGACGAGCATC
ACAAAAATCGACGCTCAAGTCAGAGGTGGCGAAACCCGACAGGACTATAAAGATACCAGGCGTTTCCCC
CTGGAAGCTCCCTCGTGCGCTCTCCTGTTCCGACCCTGCCGCTTACCGGATACCTGTCCGCCTTTCTCCCT
TCGGGAAGCGTGGCGCTTTCTCATAGCTCACGCTGTAGGTATCTCAGTTCGGTGTAGGTCGTTCGCTCCA
AGCTGGGCTGTGTGCACGAACCCCCCGTTCAGCCCGACCGCTGCGCCTTATCCGGTAACTATCGTCTTGA
GTCCAACCCGGTAAGACACGACTTATCGCCACTGGCAGCAGCCACTGGTAACAGGATTAGCAGAGCGA
GGTATGTAGGCGGTGCTACAGAGTTCTTGAAGTGGTGGCCTAACTACGGCTACACTAGAAGGACAGTAT
TTGGTATCTGCGCTCTGCTGAAGCCAGTTACCTTCGGAAAAAGAGTTGGTAGCTCTTGATCCGGCAAACA
AACCACCGCTGGTAGCGGTGGTTTTTTTGTTTGCAAGCAGCAGATTACGCGCAGAAAAAAAGGATCTCA
AGAAGATCCTTTGATCTTTTCTACGGGGTCTGACGCTCAGTGGAACGAAAACTCACGTTAAGGGATTTTG
GTCATGAGATTATCAAAAAGGATCTTCACCTAGATCCTTTTAAATTAAAAATGAAGTTTTAAATCAATCTA

AAGTATATATGAGTAAACTTGGTCTGACAGTTACCAATGCTTAATCAGTGAGGCACCTATCTCAGCGATC
TGTCTATTTCGTTCATCCATAGTTGCCTGACTCCCCGTCGTGTAGATAACTACGATACGGGAGGGCTTAC
CATCTGGCCCCAGTGCTGCAATGATACCGCGAGACCCACGCTCACCGGCTCCAGATTTATCAGCAATAAA
CCAGCCAGCCGGAAGGGCCGAGCGCAGAAGTGGTCCTGCAACTTTATCCGCCTCCATCCAGTCTATTAA
TTGTTGCCGGGAAGCTAGAGTAAGTAGTTCGCCAGTTAATAGTTTGCGCAACGTTGTTGCCATTGCTACA
GGCATCGTGGTGTCACGCTCGTCGTTTGGTATGGCTTCATTCAGCTCCGGTTCCCAACGATCAAGGCGA
GTTACATGATCCCCCATGTTGTGCAAAAAAGCGGTTAGCTCCTTCGGTCCTCCGATCGTTGTCAGAAGTA
AGTTGGCCGCAGTGTTATCACTCATGGTTATGGCAGCACTGCATAATTCTCTTACTGTCATGCCATCCGTA
AGATGCTTTTCTGTGACTGGTGAGTACTCAACCAAGTCATTCTGAGAATAGTGTATGCGGCGACCGAGTT
GCTCTTGCCCGGCGTCAATACGGGATAATACCGCGCCACATAGCAGAACTTTAAAAGTGCTCATCATTG
GAAAACGTTCTTCGGGGCGAAAACTCTCAAGGATCTTACCGCTGTTGAGATCCAGTTCGATGTAACCCAC
TCGTGCACCCAACTGATCTTCAGCATCTTTTACTTTCACCAGCGTTTCTGGGTGAGCAAAAACAGGAAGG
CAAAATGCCGCAAAAAAGGGAATAAGGGCGACACGGAAATGTTGAATACTCATACTCTTCCTTTTTCAAT
ATTATTGAAGCATTTATCAGGGTTATTGTCTCATGAGCGGATACATATTTGAATGTATTTAGAAAAATAA
ACAAATAGGGGTTCCGCGCACATTTCCCCGAAAAGTGCCACCTGACGTCTAAGAAACCATTATTATCATG
ACATTAACCTATAAAAATAGGCGTATCACGAGGCAGAATTTCAGATAAAAAAAATCCTTAGCTTTCGCTA
AGGATGATTTC
